# Authors’ Response to Peer Reviews of “Are We Sure We Fully Understand What an Infodemic Is? A Global Perspective on Infodemiological Problems”

**DOI:** 10.2196/40636

**Published:** 2022-07-21

**Authors:** Alessandro Rovetta, Lucia Castaldo

**Affiliations:** 1 R&C Research Bovezzo (Brescia) Italy

**Keywords:** communication, conspiracy, COVID-19, education, fake news, infodemic, infodemiology, mass media, public health, risk perception, science

*This is the authors’ response to peer-review reports for “Are We Sure We Fully Understand What an Infodemic Is? A Global Perspective on Infodemiological Problems.”*

## Round 1 Review

Dear Editor,

We appreciate the opportunity to review our manuscript for re-evaluation. We sincerely thank the reviewers for suggesting substantial improvements to our work. If further changes are deemed necessary, we will be more than willing to make them.

### Anonymous [1]

Comment 1: “The neologism “dismisinformation” is problematic; we commonly use “misinformation” as an umbrella term when we cannot distinguish the type of information disorder.”

Answer: Dear reviewer, we agree that the exact definition of disinformation and misinformation lacks uniformity, which can create ambiguity. For example, some authors prefer to totally separate misinformation (understood as involuntary) from disinformation (understood as voluntary) [2]. For instance, Wang et al [3] argue that “Misinformation involves information that is inadvertently false and is shared without intent to cause harm, while disinformation involves false information knowingly being created and shared to cause harm.” Moreover, O’Hair et al [4] propose the following definition: “A formal definition of dismisinformation is any message or a set of messages that represent a meaning complex discrepant from or incompatible with a sender's intent and/or a relatively informed or expert consensual evidentiary state.” We have proceeded to specify this important detail in the manuscript (please see the “Infodemiology” subsection). The added text is as follows: “Specifically, O’Hair et al formally define dismisinformation as ‘any message or a set of messages that represent a meaning complex discrepant from or incompatible with a sender's intent and/or a relatively informed or expert consensual evidentiary state.’”

Comment 2: The definition of fake news is advancing toward a specific information disorder, that is, it is not a mere simplification of phenomena (see, for instance, Molina et al).

Answer: Dear reviewer, we agree with this point. Indeed, our sentence specifies that the expression “fake news” is sometimes used as a synonym of “dismisinformation” in its broadest sense. In this regard, Wang et al [3] argue that “Although ‘fake news’ is the term that received most popular attention, it is arguably the most problematic one in terms of definitional rigour.” We have modified the paper specifying this aspect and citing the proposed reference (please see the “Infodemiology” subsection).

The added text is as follows: “To date, there is no univocal cataloging of the various types of infodemic information. For instance, Wardle et al define ‘disinformation’ as the intersection between misinformation (eg, false connection and misleading content) and malinformation (eg, leaks, harassment, and hate speech). On the contrary, Wang et al argue that when the dissemination is voluntary and takes place for malicious purposes, we speak of disinformation; otherwise (ie, when it is unintentional and accidental) we speak of misinformation. Some authors enclose both meanings in the unique term ‘dismisinformation,’ while others adopt the sometimes-criticized expression ‘fake news’… In this regard, it is essential to point out that these denominations can include false news, polarized content, satire, misreporting, commentary, persuasive information, and citizen journalism.”

Comment 3: The authors affirm that infodemics cannot exist without dismisinformation. This sentence is imprecise because information disorder also includes malinformation, fake news, and conspiracy theory. The background adopted by the authors to reflect on the presented problems can be compromised by such misconceptions.

Answer: Dear reviewer, thank you for pointing out this shortcoming. We clarified this point in the introductory section on infodemiology. It was specified to the reader that the definition proposed by O'Hair [4] includes these phenomena.

The added text is as follows: “In this paper, we will adopt the O'Hair convention. Phenomena such as malinformation and conspiracy hypotheses will therefore be included in the concept of dis-misinformation.”

Comment 4: I recommend that the authors concentrate their efforts on a specific problem, presenting a deep argumentation about the mechanisms that contribute to the success of information disorder during the pandemic.

Answer: Dear reviewer, we agree that focusing on a specific topic would increase the impact of the discussion on the individual topic. However, the purpose of this perspective is to provide a brief summary of all possible problems to consider when devising an infodemiological strategy. On the other hand, future papers may be developed to address the individual issues appropriately, starting from this general background.

### Reviewer BM [5]

Comment: Dear Authors

This paper presents a scientific and futuristic discourse on the context of infodemiology. However, I suggest arranging the content in order of importance. For example, I think the problem of predatory journals is overexplained. Moreover, the suggestion given regarding the mentioned problem is not practical, and the statements about the relationship between the editor and the referee do not seem fair. Additionally, the authors' statements about the duration of the submission review process are incomplete without an innovative result or proposal. In addition, the suggestions in the last part of the article need further explanation. Lastly, some of the items mentioned in the Abstract of the article have received little attention in the main text.

Answer: Dear reviewer, thank you for your evaluation of our paper. We have improved the abstract as suggested to make it clearer and more consistent with the content of the manuscript. In this regard, we have tried to clarify that this paper does not want to propose easy solutions but provide an overview of the problems to be faced in order to solve the infodemic issue. Indeed, we have specified that our suggestions (eg, degrees of reliability) are highly indicative and can be used as a mere starting point to then be delineated by more targeted research. The aim is that this manuscript can be read by both a specialized and lay public. Precisely for this reason, we have tried to explain scenarios (such as those of predatory publication) that are not known by those who are not in the sector but are fundamental from a communicative point of view (indeed, the public can be confused about the difference between a journal and another). Finally, we respectively disagree that the proposed solutions are not innovative: for example, at present, the communication format adopted in television shows and even newspapers does not include the presentation of evidence reliability through a specifically dedicated scale. This has given way to public figures comparing their mere personal opinions with peer-reviewed literature, generating extreme confusion in an inexperienced audience. Therefore, we strongly believe that our proposal may be a very straightforward way of limiting the disclosure of conflicting information based solely on the principle of individualistic authority. In fact, the color presentation of information is a method already widely used to determine the severity of epidemiological situations (eg, COVID-19) as it is very easy to interpret.

### Anonymous [6]

Comment: This paper is a well-written, informative study. However, it has some grammatical errors.

Answer: Dear reviewer, thank you very much for your review. We are pleased about your comment. Following your indications, we have done a grammar revision of the whole paper through the professional version of the “Grammarly” software.

### Reviewer CE [7]

Comment: This is a reasonable viewpoint/opinion paper [2]. I do not agree with everything that is being said but that is also not the goal—it is the authors’ opinion.

I do think the paper should be transferred to JMIR Infodemiology. As to the authors’ statement that “the obsessive pursuit of prestige must be drastically limited as they undermine the credibility of science,” I agree, and that also extends to obsession with the impact factor, so I hope the author follows his own advice and agrees to a transfer.

Answer: Dear reviewer, thank you very much for your review and intellectual openness. We want to stress that there is no problem in publishing our paper in a journal without an impact factor. In this regard, we specify that JMIRx Med (our first option) currently does not have impact factor.

Comment 1: It may be worth citing Mackey et al in addition to ref 1.

Answer: Dear reviewer, thank you very much for this relevant suggestion. The paper has been added as a reference.

Comment 2: Preprint servers do screen submissions, and there are different levels of screening, varying by preprint server. For example, MedRxiv implemented more strict criteria on COVID-19 compared with Zenodo, etc.

Answer: Dear reviewer, thank you for pointing out this important aspect. We have briefly discussed this information in the manuscript to provide a more complete and clear background on preprints.

The added text is as follows: “Besides, it is essential to consider that screening criteria are not uniform between the various preprints platforms: for instance, medRxiv and bioRxiv repositories operate stricter selection criteria about COVID-19 than other databases. Hence, it is also necessary to consider this aspect when evaluating the classification level.”

Comment 3: “Level of evidence” is a well-known phrase and is typically thought of in terms of study type rather than dissemination modality (ie, “systematic review” is better than “RCT,” which is better than “observational studies,” etc). If you come up with a new hierarchy—that is not directly speaking to the study type—I would suggest you come up with a new phrase or label for the type of hierarchy you are suggesting.

Answer: Dear reviewer, thank you very much for this essential clarification. We fully agree that using the term “degrees of evidence” is inappropriate as it is already adopted for a different purpose. Therefore, we have proposed the “degree of reliability,” a scale that considers both the levels of evidence and the credibility of a paper.

The added text is as follows: “Further critical issues arise when presenting sensitive information to the public: indeed, it is not just a matter of communicating the degree of evidence (eg, original article vs meta-analysis) but also its credibility (eg, publication in a predatory journal vs publication in a legitimate journal). Therefore, the public should be educated on what we have termed ‘degree of reliability’ (ie, a scale that considers both the level of evidence and the credibility of scientific works).”
